# Nitric Oxide Donor Molsidomine Positively Modulates Myogenic Differentiation of Embryonic Endothelial Progenitors

**DOI:** 10.1371/journal.pone.0164893

**Published:** 2016-10-19

**Authors:** Mario Tirone, Valentina Conti, Fabio Manenti, Pier Andrea Nicolosi, Cristina D’Orlando, Emanuele Azzoni, Silvia Brunelli

**Affiliations:** 1 School of Medicine and Surgery, University of Milano-Bicocca, Monza, Italy; 2 Division of Immunology, Transplantation and Infectious Diseases, San Raffaele Scientific Institute, Milan, Italy; 3 Division of Regenerative Medicine, Stem Cells and Gene Therapy, San Raffaele Scientific Institute, Milan, Italy; 4 MRC Molecular Hematology Unit, Weatherall Institute of Molecular Medicine, John Radcliffe Hospital, University of Oxford, Oxford, United Kingdom; Centro Cardiologico Monzino, ITALY

## Abstract

Embryonic VE-Cadherin-expressing progenitors (eVE-Cad^+^), including hemogenic endothelium, have been shown to generate hematopoietic stem cells and a variety of other progenitors, including mesoangioblasts, or MABs. MABs are vessel-associated progenitors with multilineage mesodermal differentiation potential that can physiologically contribute to skeletal muscle development and regeneration, and have been used in an *ex vivo* cell therapy setting for the treatment of muscular dystrophy. There is currently a therapeutic need for molecules that could improve the efficacy of cell therapy protocols; one such good candidate is nitric oxide. Several studies in animal models of muscle dystrophy have demonstrated that nitric oxide donors provide several beneficial effects, including modulation of the activity of endogenous cell populations involved in muscle repair and the delay of muscle degeneration. Here we used a genetic lineage tracing approach to investigate whether the therapeutic effect of nitric oxide in muscle repair could derive from an improvement in the myogenic differentiation of eVE-Cad^+^ progenitors during embryogenesis. We show that early *in vivo* treatment with the nitric oxide donor molsidomine enhances eVE-Cad^+^ contribution to embryonic and fetal myogenesis, and that this effect could originate from a modulation of the properties of yolk sac hemogenic endothelium.

## Introduction

Over the last years, the existence of different stem or progenitor cells with myogenic potential has been widely explored. In addition to the typical skeletal muscle progenitors, the satellite cells, many other multipotent and embryologically unrelated progenitors bearing potential roles in muscle differentiation and tissue repair have been identified [1]. In particular, a population of progenitor cells named mesoangioblasts (MABs) has been identified in the embryonic dorsal aorta [2]. They express markers of hemangioblastic, hematopoietic, endothelial and mesodermal lineages, and exhibit self-renewal properties and mesodermal differentiation capabilities both *in vitro* and *in vivo* [2, 3].

Using a Cre-loxP based genetic lineage tracing system, we have shown that the hemogenic endothelium in the mouse embryo can undergo mesenchymal transition and is the source of CD45^+^ progenitor cells. These are distinct from embryonic MΦs and can give rise both to hematopoietic cells and mesenchymal progenitor cells. The latter bear characteristics of embryonic MABs and are able to physiologically contribute to different mesodermal lineages in the embryo, including the skeletal muscle [4].

The ability of MABS to be easily isolated, to differentiate *in vitro* and *in vivo* into skeletal muscle, and to cross the vessel walls when transplanted [2, 5], has prompted their use in exogenous cell therapy approaches for muscle degenerative diseases, in particular in models of muscular dystrophies (MDs). MDs are a heterogeneous group of genetic diseases, characterized by a progressive and irreversible degeneration of skeletal muscle with the most severe cases leading to progressive paralysis and death. MABs have been successful in cell transplantation protocols in dystrophic animals [6–9] thus leading to an ongoing clinical trial for human Duchenne’s muscular dystrophy (DMD) patients using the human counterparts of MABs [10]. However, although encouraging, this cell therapy approach is not currently able to fully repair the structural organization and restore the function of the dystrophic muscle. Additional limitations include the high cost and the requirement to tailor the therapy for each patient given the current state-of-the-art. An alternative therapeutical approach to the cell transplantation involves endogenous stem cells which are activated following injury, but in the case of chronic degenerative disease undergo a quick exhaustion. Therefore, an optimal intervention would require the activation of endogenous myogenic stem cells and their expansion and maintenance by molecules acting on specific signaling pathways.

Several growth factors and cytokines have been shown to activate resident mesodermal or circulating stem cells. The observation that pathophysiological features of MDs are associated to an abnormal production of nitric oxide (NO) [11] has prompted studies focusing on the role of NO in muscle development and regeneration and its potential use as a therapeutic agent, either alone [12–16] or in combination with nonsteroidal anti-inflammatory (NSAID) drugs or MAB-based cell therapies [6, 17, 18].

One specific NO donor, molsidomine, was shown to slow disease progression in the absence of NSAIDs and to re-establish the functional capability of the damaged muscle, considerably ameliorating its motor activity [12]. Molsidomine treatment *per se* was able to effectively modulate the features of the inflammatory cells that infiltrate the dystrophic muscles, reducing the fibrotic scar tissue and enhancing its healing function [19] and regulating fibro-adipogenic precursor differentiation [13]. Furthermore, the beneficial effect of molsidomine could be explained by its ability to enhance the self-renewal capacity of satellite cells, thus counteracting the impoverishment of the satellite cells pool [12]. Our group has demonstrated that molsidomine has a favourable impact on embryonic myogenesis in alpha-sarcoglycan (α-SG) null mice by increasing the number of myogenic stem cells [12]. This early effect could be of great importance since it has been shown that in dystrophic muscles, stem cell depletion begins during late embryonic life [20] and experimental treatments in animal models at perinatal stages led to a significant amelioration of the dystrophic phenotype [21]. A better understanding of NO effect on different embryonic progenitors and on the molecular pathways downstream NO signaling in these cells would pave the way to design novel therapies, suitable for treating already in the early stages of the disease and could improve the outcome of other therapeutic strategies at later stages.

Here we investigated whether the positive effect of molsidomine on foetal myogenesis could arise from a modulation of the fate of endogenous embryonic MABs.

## Materials and Methods

### Animals and Treatment

Mice were housed in the SPF animal facility at our Institute and treated with the approval of San Raffaele Scientific Institutional Animal Care and Use Committee (IACUC 489, 663) in accordance with the Italian law and to the European Community guidelines. Pregnant females were euthanised (CO_2_ induced narcosis prior to cervical dislocation) prior to embryos recovery in accordance with the European Community guidelines and with the approval of San Raffaele Scientific Institutional Animal Care and Use Committee (IACUC 489, 663) in accordance with the Italian law.

The transgenic mice that have been used in this study are: Cdh5-CRE^ERT2^ [22]; R26R [23]; R26R-EYFP [24]. Mice were kept as heterozygous and were genotyped as in [4].

Cre recombination was induced by injecting 2mg/25g body weight of Tamoxifen (TAM) (T5648, Sigma-Aldrich, Saint Louis, MO, USA; 10 mg/ml in corn oil) intra-peritoneally (IP) into pregnant females. Staging of early embryos (E9.5) was performed by counting the pairs of somites (E9.5: 18-26sp). For E12.5 and E15.5 embryos we considered that fertilization took place at 6 a.m.

Standard diet (STD) or a diet containing 3 mg/kg of (1-ethoxy-N-(3-morpholino-5-oxadiazol-3-iumyl)-methanimidate (molsidomine) was set based on the daily food intake measured for these animals as in [12]. The experimental groups did not display significant differences in food intake and weight gain.

### Embryos and foetuses

For histological analysis, dissected E12.5-E15.5 embryos were fixed for 2–3 hours with a 4% solution of paraformaldehyde (PFA) in PBS at 4°C. After that, embryos were washed in PBS and dehydrated/cryoprotected with passages in PBS solutions with increasing sucrose concentration (10% for 1 hour, 20% for 1 hour, 30% overnight). Embryos were subsequently embedded in OCT and sectioned using a Leica 1850UV cryostat (8μM sections were made).

### Immunofluorescence and antibodies

Immunofluorescence on frozen section was carried out as in [4]. The antibodies used are listed in S1 Table. A minimum of 6 embryos were analyzed for each condition. Histological quantifications were done by counting 20 fields (20x and/or 40x) for each data point.

### Flow cytometry

eVE-Cad^+^ derived cells were handled as in [4]. Cell sorting and FACS analysis were performed using the MoFLo XDP system (Beckman Coulter, Inc., Brea, CA, US) and LSR Fortessa or FacsCANTO (BD Bioscience, Bedford, MA, USA) respectively. For E9.5 embryos 6–10 embryos and yolk sacs (YS) were pooled for each experiment. For E12.5 embryos 2 embryos were pooled for each experiment. Doublets were excluded by gating on physical parameters; dead cells were gated out based on Hoechst 33258 uptake (H3569, Invitrogen, Carlsbad, CA, USA) or 7-Aminoactinomycin D (A9400, Sigma-Aldrich, Saint Louis, MO, USA). We used fluorescence minus one (FMO) controls and single stain controls to set the position of the gates. Isotype and FMO controls are shown in S1 Fig. Data were analyzed using FlowJo software (TreeStar). The antibodies used are listed in S2 Table.

### Quantitative Real-Time PCR

EYFP^+^ cells sorted from E9.5 or E12.5 embryos were processed using ReliaPrep™ RNA Cell Miniprep System (Z6011, Promega, Milan, Italy). Reverse transcription (RT) was done using the High-Capacity cDNA Reverse Transcription Kit (4368814, Applied Biosystems, Foster, CA, USA). qRT-PCR analysis was carried out using LightCycler 480 (Roche, Basel, Switzerland) or the 7900HT FAST (Applied Biosystems, Foster, CA, USA) Real-Time PCR detection systems. cDNAs were amplified using the GoTaq qPCR Master Mix and the Hot Start Polymerase (A6001, Promega, Milan, Italy). Primer sequences are listed in S3 Table. CT values greater than 35 were considered as negative. Data points were analyzed in triplicate. Quantification was performed using the comparative C_T_ method. Internal controls: 28S or cyclophilin A.

### Gene expression profiling

For gene expression profiling analyses, sorted EYFP^+^ cells from E9.5-E10.5 Cdh5-CreER^T2^; R26R-EYFP embryos were processed using ReliaPrep™ RNA Cell Miniprep System (Z6011, Promega, Milan, Italy). cDNA was prepared using RT^2^ First Strand Kit (330401 Qiagen, Hilden, Germany). Analyses were done using the Mouse Stem Cell Signaling RT2 Profiler™ PCR Arrays (PAMM-047Z, Qiagen, Hilden, Germany) according to manufacturer’s instructions.

### Image acquisition and manipulation

Fluorescent images were taken using the following microscopes: Leica TCS SP2 Laser Scanning Confocal or Zeiss LSM 710 Confocal Microscope.

Images were processed using Adobe Photoshop CS6 and Adobe Illustrator CS6.

### Statistical analysis

Data were analyzed with Microsoft Excel 14.1.0 and GraphPad Prism 6 and was plotted as mean ± SD or mean ± SEM. To evaluate statistical significance unpaired two-tailed Student’s *t*-tests were used assuming equal variance.

## Results

### Molsidomine treatment affects the specification of VE-Cad^+^ derived progenitors

To study the effect of nitric oxide on embryonic endothelial derived progenitors, we exploited a transgenic mouse line expressing a tamoxifen inducible (CRE-ER^T2^) Cre recombinase under the control of VE-Cadherin regulatory sequences, Cdh5-Cre^ERT2^ [4]. These mice were crossed with R26R-EYFP Cre reporter mice in order to obtain double transgenic embryos. We induced Cre recombination by injecting tamoxifen (TAM) IP into the pregnant mother at E8.5, to genetically label VE-Cad expressing cells and their progeny with EYFP. Efficiency and specificity of the recombination were consistent with our previous reports [4].

The NO-releasing drug molsidomine was administered at the beginning of the pregnancy and the treatment continued until the recovery of the embryos/fetuses as in [12]. Control embryos were collected from mice fed with a standard diet.

Firstly, we evaluated by FACS analysis the effect of molsidomine treatment on the total number of embryonic endothelial progenitors (eVE-Cad^+^). We prepared single cell suspensions from embryos collected at E12.5, a time at which we previously showed that EYFP^+^ labeled cells start to become heterogeneous and when MAB-like cells can be found abundantly in the mesenchyme [4].

**Fig 1:**
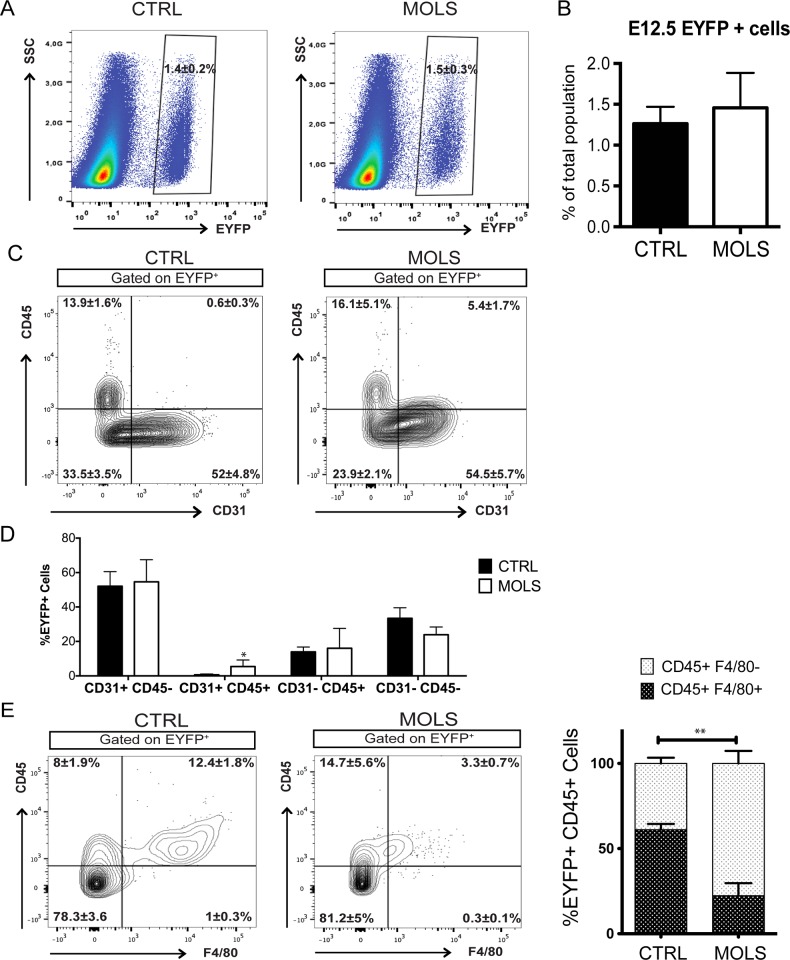
Effect of molsidomine treatment during embryogenesis on eVE-Cad^+^ derived cells. A) EYFP^+^ gating strategy used for FACS analysis of single cells suspensions from E12.5 Cdh5-CREERT2; R26R-EYFP embryos (minus the head and fetal liver) B) Graph representing the percentage of EYFP^+^ cells obtained by FACS analysis of single cell suspensions from E12.5 embryos (At least 5 embryos per group, n = 3 independent experiments). Data are represented as mean ± S.E.M. C) FACS analysis of E12.5 embryos cells showing the expression of CD45 and CD31 within the EYFP^+^ subset. D) Graph summarizing the percentage of the different populations shown in (C), obtained with at least 4 control and 5 molsidomine-treated embryos, n = 3 independent experiments. Data are expressed as mean ± S.E.M; *p<0.05 (MOLS vs CTRL). E) FACS analysis on E12.5 embryos showing the expression of CD45 and F4/80 within the EYFP^+^ subset. F) Graph summarizing the percentage of F4/80^+^ cells within the EYFP^+^CD45^+^ subset obtained with 4 control and 5 molsidomine-treated embryos, n = 3 independent experiments. Data are expressed as mean ± S.E.M; **p<0.01 (MOLS vs CTRL).

We observed that the percentage of EYFP^+^ cells on the total cell population was not significantly changed by molsidomine treatment (1.4±0.2% in CTRL embryos vs 1.5±0.3% in molsidomine embryos) (Fig 1A and 1B). This result indicates that the treatment with a nitric oxide donor drug does not induce the expansion of embryonic endothelial progenitors and/or their progeny.

Concomitantly with this analysis, we evaluated the effect of molsidomine treatment on the distribution of EYFP^+^ cells in the two main subpopulations at this developmental stage: the endothelial (CD31^+^CD45^-^) and the hematopoietic (CD45^+^CD31^-^).

We observed no significant difference in the fraction of CD31^+^ CD45^-^ cells within the EYFP^+^ population (52±4.8% vs 54.5±5.7% in CTRL and molsidomine embryos, respectively) after molsidomine treatment. The EYFP^+^ CD45^+^CD31^-^ hematopoietic subset was also not significantly changed (16.1±5.1% in treated embryos vs 13.9±1.6% in control embryos) (Fig 1C and 1D). However, in molsidomine treated embryos we detected CD31^+^ cells also expressing CD45^+^, a population that we did not observe in control animals (5.4±1.7% in molsidomine embryos vs 0.6±0.3 in control embryos, p<0.05%) (Fig 1C and 1D).

We also determined the proportions of the macrophage (F4/80^+^) and non-macrophage (non-MΦ F4/80^-^) subsets in the hematopoietic population, since in our previous work we showed that, amongst the EYFP^+^ population, mesoangioblast-like cells belonged to the CD31^-^ CD45^+^ F4/80^-^ subpopulation [4].

In molsidomine treated embryos we observed an increased trend in the number of CD45^+^F4/80^-^ cells within the EYFP^+^ population (14.7±5.6% in treated embyos vs 8±1.9% in control embryos) and a corresponding significant decrease in the number of MΦ CD45^+^F4/80^+^ (12.4±1.8% in control embryos vs 3.3±0.7% in molsidomine treated embryos, p<0.01) (Fig 1E). This increase was even more evident by analyzing the percentage of the non-MΦ F4/80^-^ inside the CD45^+^ population, higher in molsidomine treated embryos (77.7±7.3% vs 38.9±3.4% in control embryos, p<0.01) (Fig 1F).

### Molsidomine treatment increases the myogenic differentiation of eVE-Cad^+^ progenitor cells

We have previously demonstrated that cells expressing VE-Cad at E8.5 and/or their progeny physiologically contribute to the myogenic lineage without differentiating through a somitic intermediate [4].

We therefore examined how molsidomine influences the contribution of eVE-Cad^+^ derived progenitors to the development of the skeletal muscle at two different timepoints, E12.5 and E15.5, corresponding respectively to the times of establishment of embryonic and foetal myogenesis [25].

**Fig 2:**
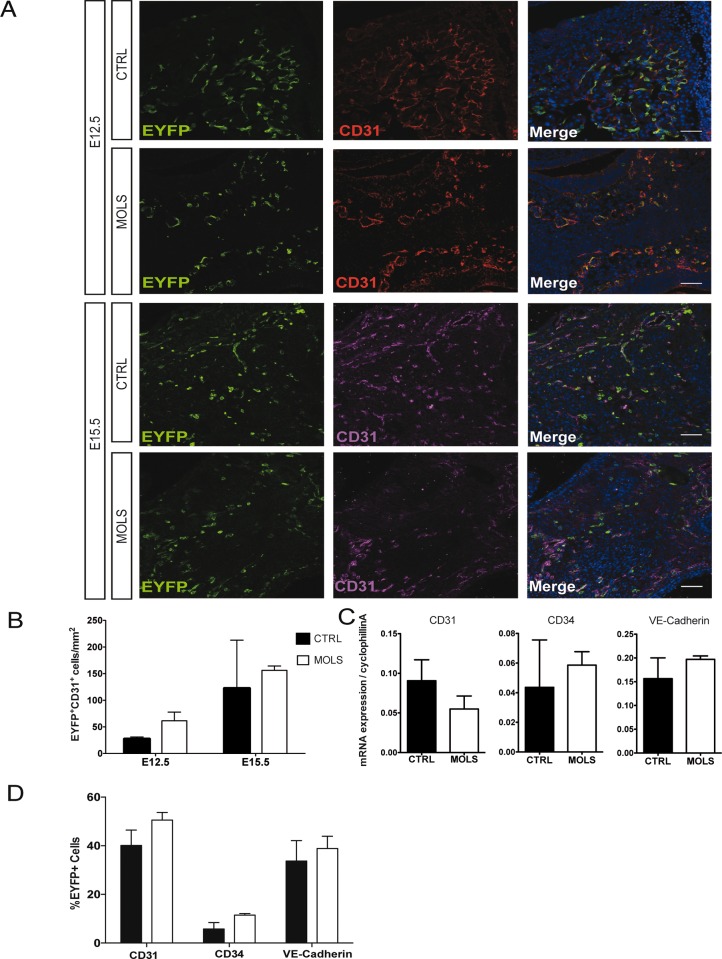
Molsidomine treatment during embryonic development does not affect eve-cad^+^ derived cells contribution to angiogenesis. A) Immunofluorescence (IF) using anti-EYFP and anti-CD31-specific antibodies on transverse sections of Cdh5-CREERT2;R26R-EYFP E12.5 embryos and E15.5 fetuses, untreated (CTRL) and treated with molsidomine (MOLS). Nuclei are stained with Hoechst. Scale bars: 50 um. B) Graph represents the quantification of the number of EYFP^+^/CD31^+^ cells in the embryo, performed by counting cells in at least 20 fields in different areas (20x and/or 40x) for each data point. Data are expressed as mean± S.E.M. (n = 6 embryos). C) qRT-PCR analysis on EYFP^+^ cells freshly sorted at E12.5. Gene expression data are relative to cyclophilin A. D) Graph summarizing the percentage of CD31^+^, CD34^+^, VE-Cad^+^ cells within the EYFP^+^ populations obtained with 6 control and 5 molsidomine-treated embryos, n = 3 independent experiments. Data are expressed as mean± S.E.M. (n = 4 embryos per group).

We first evaluated by immunofluorescence analysis the distribution of endothelial EYFP^+^ CD31^+^ cells and the extension of the endothelial network in control and molsidomine treated embryos. At E12.5 we observed no changes in vascularity and the distribution of EYFP^+^ CD31^+^ cells was comparable in the control and treated embryos (Fig 2A and 2B). This was also the case at E15.5 (Fig 2A and 2B). To further verify this, we performed a transcriptional analysis of eVE-Cad^+^ derived cells at E12.5, to evaluate how and to what extent the treatment with molsidomine alters endothelial gene expression.

We extracted RNA of EYFP^+^ cells sorted from E12.5 molsidomine-treated and control embryos (using the same gating strategy depicted in Fig 1A and in [4] and we performed qRT-PCR analysis evaluating the expression of a panel of endothelial genes (Fig 2C). Consistently with the immunofluorescence analysis, all of these genes (CD31, CD34, VE-Cadherin) were expressed at similar levels in control and treated embryos. FACS analysis using antibodies specific for CD31, CD34, VE-Cadherin (Fig 2D and S2 Fig) confirmed these results, suggesting that NO does not significantly alter endothelial differentiation of eVE-Cad^+^-derived progenitors.

**Fig 3:**
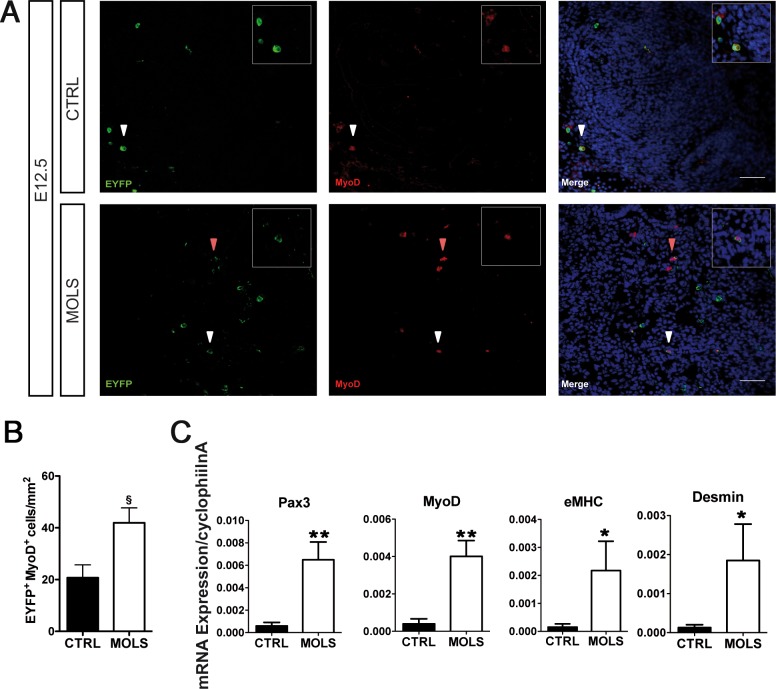
Molsidomine treatment during embryonic development increases eve-cad^+^ derived cells contribution to early myogenesis. A) Immunofluorescence (IF) using anti-EYFP and anti-MyoD-specific antibodies on transverse sections of Cdh5-CREERT2;R26R-EYFP E12.5 embryos, untreated (CTRL) and treated with molsidomine (MOLS). Nuclei were stained with Hoechst. Arrowheads indicate EYFP^+^ MyoD^+^ single-nucleated myoblasts. Inset in panels represent a 5x magnification of the cells indicated by the white arrowhead. Scale bars: 50 um; B) Graph representing the quantification of the number of EYFP^+^ MyoD^+^, cells in the embryo, performed by counting cells in at least 20 fields in different areas (20x and/or 40x) for each data point. Data are expressed as mean± S.E.M. ^§^p = 0,06; MOLS vs CTRL (n = 4 embryos per group). C) qRT-PCR analysis of EYFP^+^ cells freshly sorted at E12.5 using primers specific for Pax3, MyoD, Desmin and MyHC. Gene expression data are relative to cyclophilin A. Data are expressed as mean± S.E.M. **p≤0,01;*p≤0,05, MOLS vs CTRL (At least 6 embryos per group, n = 3 independent sorting experiments)

We next examined the contribution of eVE-Cad^+^ progenitors to embryonic and foetal myogenesis. We first evaluated the distribution of EYFP^+^ cells expressing the myogenic determination marker MyoD (Fig 3A). At E12.5 we could detect an increase in the number of EYFP^+^ myoblasts (EYFP^+^ MyoD^+^ cells) in sections of molsidomine treated embryos compared to control ones (Fig 3A and 3B). This was in agreement with the qRT-PCR analysis of EYFP^+^ cells sorted from E12.5 molsidomine-treated and control embryos showing that in EYFP^+^ cells from molsidomine-treated embryos MyoD expression was upregulated (Fig 3C). Moreover, Pax3, Desmin and eMHC, other markers of embryonic and foetal myogenesis, were also upregulated in EYFP^+^ cells derived from molsidomine treated embryos.

**Fig 4:**
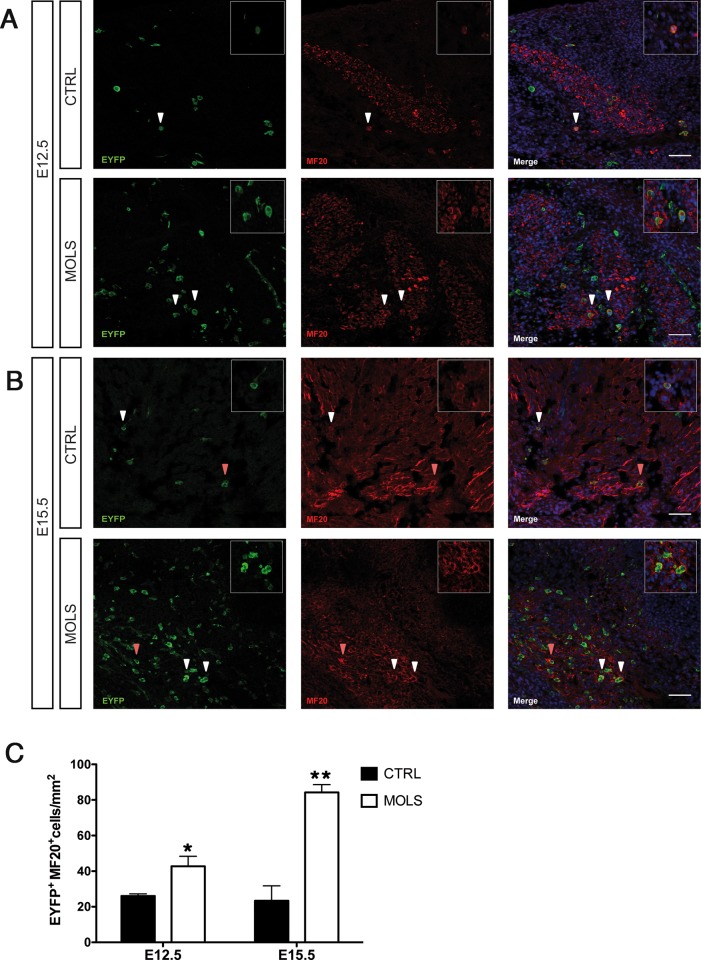
Molsidomine treatment during embryonic development increases eVE-Cad^+^ derived cells contribution to MF20^+^ myotubes. A) Immunofluorescence (IF) using anti-EYFP and MF20 specific antibodies on transverse sections of Cdh5-CREERT2;R26R-EYFP E12.5 embryos untreated (CTRL) and treated with Molsidomine (MOLS). Nuclei were stained with Hoechst. Arrowheads indicate EYFP^+^MF20^+^ single-nucleated myocytes. Inset in panels represent a 5x magnification of the cells indicated by the white arrowhead. Scale bars: 50 um; B) Immunofluorescence (IF) using anti-EYFP and anti-MF20-specific antibodies on transverse sections of Cdh5-CREERT2;R26R-EYFP E15.5 fetuses untreated (CTRL) and treated with molsidomine (MOLS). Nuclei were stained with Hoechst. Arrowheads indicate EYFP^+^MF20^+^ single-nucleated myocytes/myofibers. Inset in panels represent a 5x magnification of the cells indicated by the white arrowhead. Scale bars: 50 um. C) Graph representing the quantification of the number of EYFP^+^/MF20^+^ cells per mm^2^ in E12.5 and E15.5 embryos, performed by counting cells in at least 20 fields in different areas (20x and/or 40x) for each data point. Data are expressed as mean± S.E.M. *p≤0,05; **p≤0,005 MOLS vs CTRL (n = 4 embryos per group).

We then evaluated by immunofluorescence the expression of MyHC, a marker of differentiated myogenic cells (using the antibody MF20). At both E12.5 and E15.5, we could detect an increase in the number of EYFP^+^ myoblasts (MyHC^+^ cells) and myotubes in sections of molsidomine treated embryos compared to control ones (Fig 4A–4C). At E12.5, the number of labeled myoblasts/myotubes was approximately 1.5 times higher in molsidomine treated embryos compared to control, while at E15.5 this increase reached almost 4-fold in treated embryos (Fig 4B and 4C).

Therefore, we could conclude that molsidomine treatment increases eVE-Cad^+^ derived contribution to skeletal muscle development.

### Molsidomine treatment modulates the properties of VE-Cad^+^ hemogenic endothelium

We have previously demonstrated that embryonic MABs originate from a population of cells with mesodermal potency co-expressing hematopoietic and mesodermal markers and arising from extraembryonic VE-Cad^+^ hemogenic endothelial cells in both YS and placenta [4]. The emergence of these cells from extraembryonic hemogenic endothelium occurs in a very limited temporal window, indicating that extra-embryonic and embryonic hemogenic endothelia have a different timing of activity and possibly distinct biological characteristics, implying diverse responsiveness to distinct molecules and signaling pathways.

To investigate differences between EYFP^+^ populations labeled in different endothelial compartments, which include hemogenic endothelium, we first profiled eVE-Cad^+^ derived cells from YS and embryo proper in basal conditions by gene expression analysis, focusing on a panel of genes mainly involved in stem cell-related signaling pathways.

**Fig 5:**
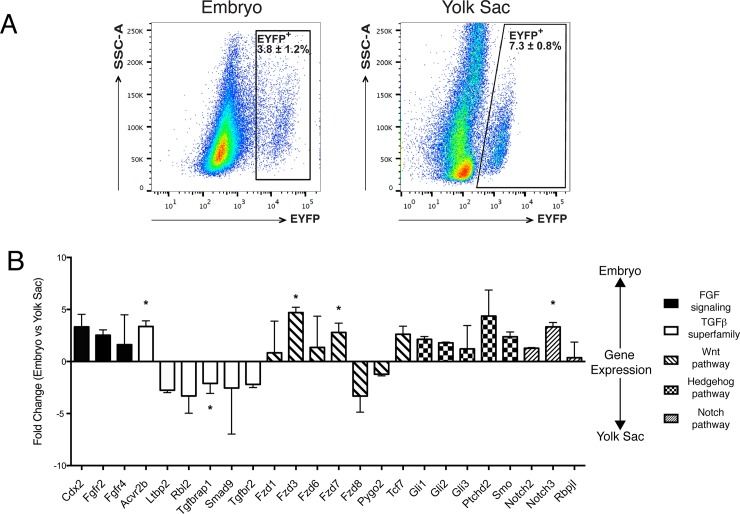
E9.5 eVE-Cad^+^ cells in the embryo and YS express a non-.overlapping set of genes. A) Gating strategy employed to isolate EYFP^+^ cells from E9.5 Cdh5-CREER^T2^;R26R-EYFP embryo proper and yolk sac by fluorescence activated cell sorting (FACS). Cre recombination was induced with TAM 24 hours before collection. Values are expressed as mean ± S.D. n≥3 independent experiments. B) EYFP^+^ cells were FACS sorted from E9.5 Cdh5-CREER^T2^;R26R-EYFP embryo and yolk sac. Cre recombination was induced with TAM 24 hours before collection. A Real-Time PCR-based array analysis was performed. Positive fold changes represent genes more expressed in the embryo respect to the YS; negative fold changes represent the opposite. At least 10 embryos and yolk sacs were pooled. Values are expressed as mean± S.E.M. *p≤0,05. n = 3 independent sorting experiments.

We sorted EYFP^+^ cells from E9.5 embryo proper and YS with Cre induction at E8.5. From previous data [4] the majority of these cells are endothelial cells (ECs) (Fig 5A). By comparing the gene expression profile of eVE-Cad^+^ derived cells from embryos or YS at E9.5 we found that groups of genes involved in the FGF, Wnt, Hedgehog and Notch signaling pathways had a higher expression in embryonic compared to YS eVE-Cad^+^derived cells (Fig 5B). In particular, Fzd3 and Notch3 were significantly increased in the embryo proper. Of those supergroups, only one gene (*Fzd8*) was more expressed in YS cells. Amongst TGF-β related genes, only Acvr2b was more expressed in the embryo proper, while the others, in particular Tgfbrap1, were upregulated in the YS at E9.5. These data indicate that E9.5 YS ECs are distinct from the endothelium in the embryo proper, possibly reflecting functional differences.

**Fig 6:**
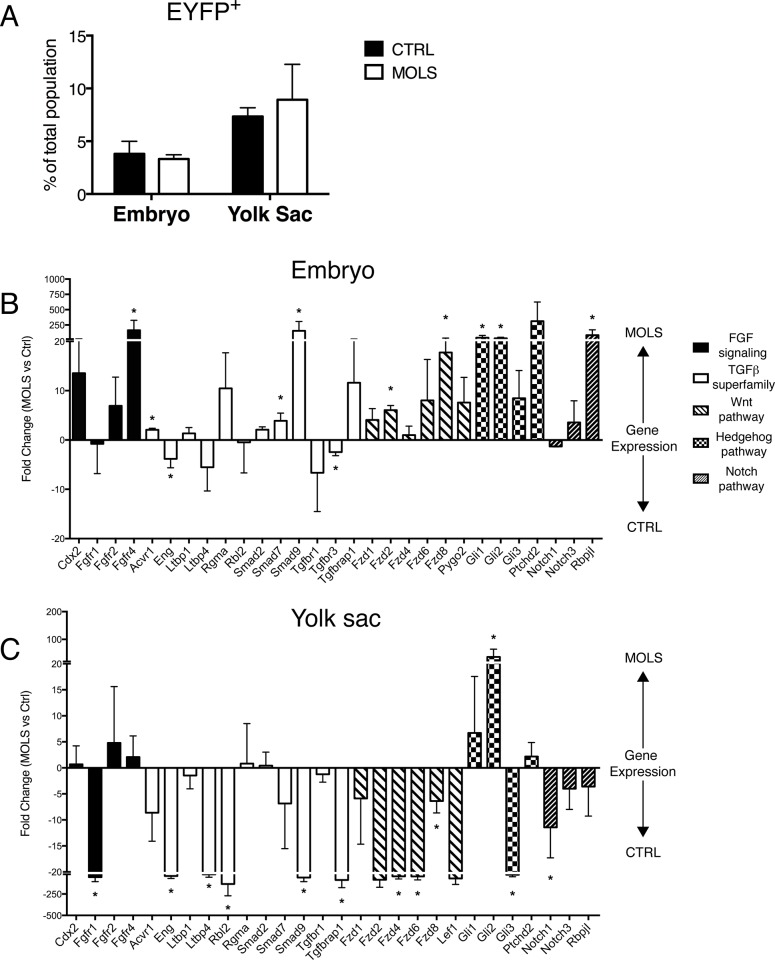
Molsidomine treatment modulates the gene expression signature of eVE-Cad^+^ cells. A) Graph summarizing the percentage of EYFP^+^ cells in molsidomine-treated (MOLS) and control (CTRL) E9.5 embryos and yolk sacs with Tamoxifen induction at E8.5. Values are expressed as mean ± S.D. n≥2 independent experiments. B-C) EYFP+ cells were FACS sorted from E9.5 Cdh5-CREER^T2^;R26R-EYFP molsidomine-treated and control embryos and yolk sacs. Cre recombination was induced with TAM 24 hours before collection. A Real-Time PCR-based array analysis was performed. Positive fold changes represent genes more expressed in molsidomine-treated (MOLS) embryos (B) and yolk sacs (C); negative fold changes represent genes more expressed in control (CTRL) embryos (B) and yolk sacs (C). At least 10 embryos and yolk sacs were pooled. Values are expressed as mean± S.E.M, *p≤0,05. n = 3 independent sorting experiments.

We have shown that molsidomine treatment could affect the myogenic differentiation of MABs/eVE-Cad^+^ cells while not changing the number or distribution of endothelial cells. We therefore reasoned that nitric oxide could directly modulate properties of different hemogenic endothelia, thereby affecting the nature of specific embryonic endothelial or hematopoietic populations, some of which physiologically contribute to myogenesis. To evaluate whether molsidomine differentially acted on yolk sac and embryonic eVE-Cad^+^ cells, we next isolated EYFP^+^ cells from control and molsidomine treated YS and embryo proper at E9.5. The percentage of EYFP^+^ cells was comparable between molsidomine treated and control embryos (Fig 6A). We then compared expression levels of the above mentioned genes in the YS and embryo proper of molsidomine-treated and control embryos (Fig 6B and 6C). In general, molsidomine treatment led to an upregulation of all these genes in the embryo proper and a downregulation in the YS, with few exceptions. In particular, the expressions of Gli2, and to a lesser extent Gli1, were higher in molsidomine- treated YS, while *Eng* and *Tfgbr3* were more expressed in the control embryo proper.

These data suggest that molsidomine treatment differentially modulates the functional properties of yolk sac and embryonic eVE-Cad^+^ cells in early embryogenesis, thus potentially influencing the fate of their progeny.

## Discussion

Several studies have clearly shown that NO administration in animal models, both alone or in combination with NSAIDs, significantly improves muscle regeneration and ameliorates the dystrophic phenotype by slowing down disease progression [6, 18, 26]. Based on this, several Phase I clinical trials have been launched and resulted in successful outcome [27, 28].

The mechanism by which NO exerts its positive effect on muscle regeneration is likely to be due to activity at multiple levels. In particular, several studies suggest that NO can influence the function of endogenous myogenic progenitors and of other cells involved in muscle development and regeneration [29].

Here we show that the NO donor molsidomine is able to modulate the differentiation, and possibly the commitment, of embryonic hemogenic endothelium-derived eVE-Cad^+^ cells, including the *in vivo* counterparts of embryonic MABs.

MABs are progenitors with physiological myogenic potential, found in embryonic and adult vessels. They have been successfully used in preclinical MD models of systemic cell therapy, leading to a significant structural and functional recovery of the skeletal muscle tissue [1, 6, 8, 9]. Despite their recent use in clinical trials, in-depth studies on their developmental origin, physiological role and biological characteristics are still missing. In particular, the fine understanding of mechanisms guiding the fate choice of these endogenous progenitors could help to identify novel ways for their *in vivo* manipulation, not only in adult life, but during embryonic and fetal development. These findings will bear great therapeutic relevance as stem cell loss in dystrophic muscles begins as early as late embryogenesis in affected individuals [20].

To date the role of NO in early embryogenesis and myogenesis has been poorly investigated. It has been shown that molsidomine treatment of α-SG null pregnant mice has beneficial effects during embryonic myogenesis, with recovery of Pax7^+^ cells in treated embryos compared to untreated dystrophic ones [12]. Additionally, recent studies have demonstrated that NO promotes embryonic myogenesis in the chicken embryo through upregulation of genes involved in myogenic differentiation [30]. However, no effect on myogenic determination genes, such as MyoD and Myf5, was found in mouse models, highlighting possible differences between mouse and chicken development. NO stimulates the proliferation of satellite cells via signaling pathways that require Vangl2 and cGMP, thus favoring muscle regeneration by counteracting the exhaustion of the satellite cells pool observed during repetitive acute and chronic damage [12]. Moreover, through a cGMP-independent pathway, NO leads to downregulation of the peroxisome proliferator-activated receptors gamma (Pparγ1) expression in fibro-adipogenic precursors (FAPs) in the skeletal muscle, therefore inhibiting their differentiation and reducing adipose tissue deposition and fibrotic scar formation [13]. The effect of NO on MABs has been investigated only on transplanted cells in the adult muscle [6, 31] but never on the endogenous pool of progenitors cells.

Here we demonstrated that NO treatment during embryogenesis results in a greater contribution of eVE-Cad^+^-derived cells (embryonic MABs) to both embryonic and foetal myogenesis. Immunofluorescence analysis showed a higher percentage of EYFP-labeled myoblasts and myotubes in molsidomine treated embryos and gene expression profiling revealed higher expression levels of myogenic genes in eVE-Cad^+^-derived cells compared to treated embryos. Importantly, molsidomine effect on myogenic differentiation of embryonic MABs appears to be specific, since we did not detect any significant difference in the number or distribution of eVE-Cad^+^-derived endothelial cells and in the expression levels of endothelial specific genes. This is in agreement with *in vitro* data showing that nitric oxide is not required for endothelial differentiation of embryonic stem cells until late stages [32].

Embryonic MABs originate from eVE-Cad^+^-derived CD45^+^ cells, that are distinct from F4/80^+^ macrophages and have the ability to differentiate into both hematopoietic cells and mesenchymal progenitor cells [4]. Here we show that molsidomine-treated embryos contain an increased number of CD45^+^ non-MΦ eVE-Cad^+^ derived cells relative to the controls, as well as an increased number of eVE-Cad^+^ CD31^+^ CD45^+^ cells, the latter possibly representing a more transitional population [4]. These results suggest that NO may act not only on myogenic differentiation of MABs, but during the early specification of MAB progenitor cells. The increase in CD45^+^ non-MΦ eVE-Cad^+^ derived cells could result from a modulation of the activity of hemogenic endothelium. Accordingly, nitric oxide has been shown to positively modulate hematopoiesis by upregulating Runx1 in the hemogenic endothelium [33, 34]. However, recent evidence has suggested that hemogenic endothelial cells are not an homogenous population [35, 36]. We have previously shown that the timing of activity of extra-embryonic and embryonic hemogenic endothelia is different, possibly due to distinct biological characteristics [4]. In particular, the YS is the source of multiple progenitors emerging independently of the embryo proper. These include erythro-myeloid progenitors (EMPs) later generating tissue-resident macrophages which persist until adult life [37], and the first immune-restricted cells with lymphoid and myeloid potential, appearing before hematopoietic stem cells (HSCs) [38]. At least some of these progenitors, including embryonic MABs [4], originate from yolk sac hemogenic endothelium [36]. This exclusive potency of extra-embryonic hemogenic endothelium implies a specific sensitivity to different signaling pathways. Interestingly, very little is known about the nature and function of different endothelial populations in the early embryo. We have focused on a specific panel of genes that has allowed us to highlight significant differences between ECs of YS and embryo proper, and to point out how NO can modulate these differences.

Definitive hemopoiesis in the AGM region, but not in the YS is Notch-dependent [39–41]. Indeed, we found that genes belonging to the Notch pathway are upregulated in the embryonic endothelium, suggesting that it is intrinsically responsive to these signals. Molsidomine treatment appears to downregulate Notch1 in the YS while Rbpj1 is upregulated in the embryo proper. The effect of molsidomine on the other pathways that we investigated shows a similar trend, with genes in the embryo proper being upregulated while their expression is reduced in the YS.

Hedgehog has been described to play a role in AGM hemopoiesis in zebrafish [42, 43]. In particular, reciprocal BMP-hedgehog gradients in the dorsal aorta appear to be critical for HSC emergence [43], while the involvement of Shh signalling in mouse hemopoiesis is less clear [44–47]. Here we show that several components of the Shh pathway are more abundantly expressed in the embryo proper in respect to the YS, while some members of the BMP/TGF pathway are more expressed in the YS. The latter are indeed known to play important roles also in the extraembryonic and early gastrulating embryo [48, 49]. Interestingly, molsidomine treatment decreases TGF/BMP activation mainly in the YS, while increasing Shh signalling in both embryo proper and YS, with the exception of the Gli3 gene.

BMP pathway also crosstalks with FGF signaling in early hemopoiesis and endothelial differentiation. It has been shown in the zebrafish model that FGF is required for the specification of aortic hemogenic endothelium via repression of BMP signaling [50]. In our control embryos, FGF signaling components are more expressed in the embryo proper. Interestingly, molsidomine treatment induces upregulation of FGF genes in the embryo, while at least Fgfr1 is highly reduced in the YS, while, as mentioned above BMP genes are downregulated mainly in the yolk sac. Moreover it has recently been reported in zebrafish that TGF/BMP pathway also interacts with the Notch pathway to orchestrate the specification of hemogenic endothelium [51]. This suggests a complex signaling network through which molsidomine may influence eVE-Cad^+^ cell fate.

Wnt signaling appears to be critical for both primitive and definitive hemopoiesis [36, 52]. *Fzd8*, the only Wnt related gene more expressed in the YS, is known to play important roles in the extraembryonic tissue and early gastrulating embryo. Molsidomine treatment differentially affects the expression of Frizzled receptors in YS and embryo proper. Again, molsidomine upregulated genes involved in this pathway in the embryo proper, while downregulating them in the YS.

We speculate that the distinct potency of yolk sac and embryonic hemogenic endothelia could be at least in part derived from differential responsiveness to extrinsic signals, such as nitric oxide. Interestingly, yolk sac EMPs still emerge in absence of circulation [36] suggesting that hemogenic endothelium in the yolk sac might have different requirements for nitric oxide signaling, as well as YS derived multipotent progenitors. This would explain the different behavior that we observed in the embryo and yolk sac ECs upon molsidomine treatment and why the endothelial differentiation of eVE-Cad^+^ derived cells was not largely changed.

The crosstalk of NO with other signaling pathways in the embryo is mostly unexplored and this is the first evidence of its broad effect on the physiology of embryonic endothelial progenitor derived cells. More specific studies will be required to get additional insights into the role of NO in the fate commitment of endothelial progenitors and to unravel the role of its downstream pathways and their crosstalk in the differential nature and developmental fate of YS and embryonic ECs. These studies may ultimately pave the way on new approaches to manipulate cell fate in the embryo and at the same time develop new therapeutic approaches.

## Conclusions

In this study, using a mouse model we demonstrate that treatment with the NO donor molsidomine during pregnancy modulates the fate of embryonic endothelial-derived progenitors. Notably, while not affecting endothelial network formation, molsidomine treatment enhances the contribution of endothelial-derived progenitors to embryonic and fetal myogenesis. We also provide evidence that molsidomine treatment affects several important signaling pathways in the embryonic endothelium, possibly resulting in the expansion of a CD45^+^ non-MΦ eVE-Cad^+^ population, that we have previously shown to include mesoangioblast-like cells. These findings may help explain the biology behind the beneficial effect of NO in embryonic myogenesis and could in the future have therapeutic relevance for the early *in vivo* and *ex vivo* treatment of muscular dystrophy.

## Supporting Information

S1 Fig Isotype and Fluorescence Minus One (FMO) controls for FACS staining. A) Anti-IgG2b,k- PE and Anti-IgG2a,k-APC isotype controls for FACS analysis of E12.5 embryos. B) Representative density plots showing FMO controls for FACS analysis of E12.5 cells. Plots show gating of CD31 and CD45 within the EYFP^+^ population. C) Representative density plots showing FMO controls for FACS analysis of E12.5 cells. Plots show gating of CD31 and F4/80 within the EYFP^+^ population.
(TIF)

S2 Fig Effect of molsidomine treatment on eVE-Cad^+^ cells during vascular embryogenesis. A-C) Representative FACS plots of E12.5 embryos showing the percentage of (A) CD31, (B) CD34 and (C) VE-Cadherin within the EYFP^+^ subset in control and molsidomine treated embryos. Data are expressed as mean ± S.E.M. (At least n = 3 embryos per group).
(TIF)

S1 Table List of antibodies used for Immunofluorescence. (PDF)

S2 Table List of antibodies used for FACS analysis and FACS sorting. (PDF)

S3 Table List of primers used for quantitative real-time PCR. (PDF)
