# Supplementary figures and images for: Nitric Oxide Donor Molsidomine Positively Modulates Myogenic Differentiation of Embryonic Endothelial Progenitors
Source: PLoS One. 2016 Oct 19;11(10):e0164893. doi: 10.1371/journal.pone.0164893 (PMC5070765; doi:10.1371/journal.pone.0164893)

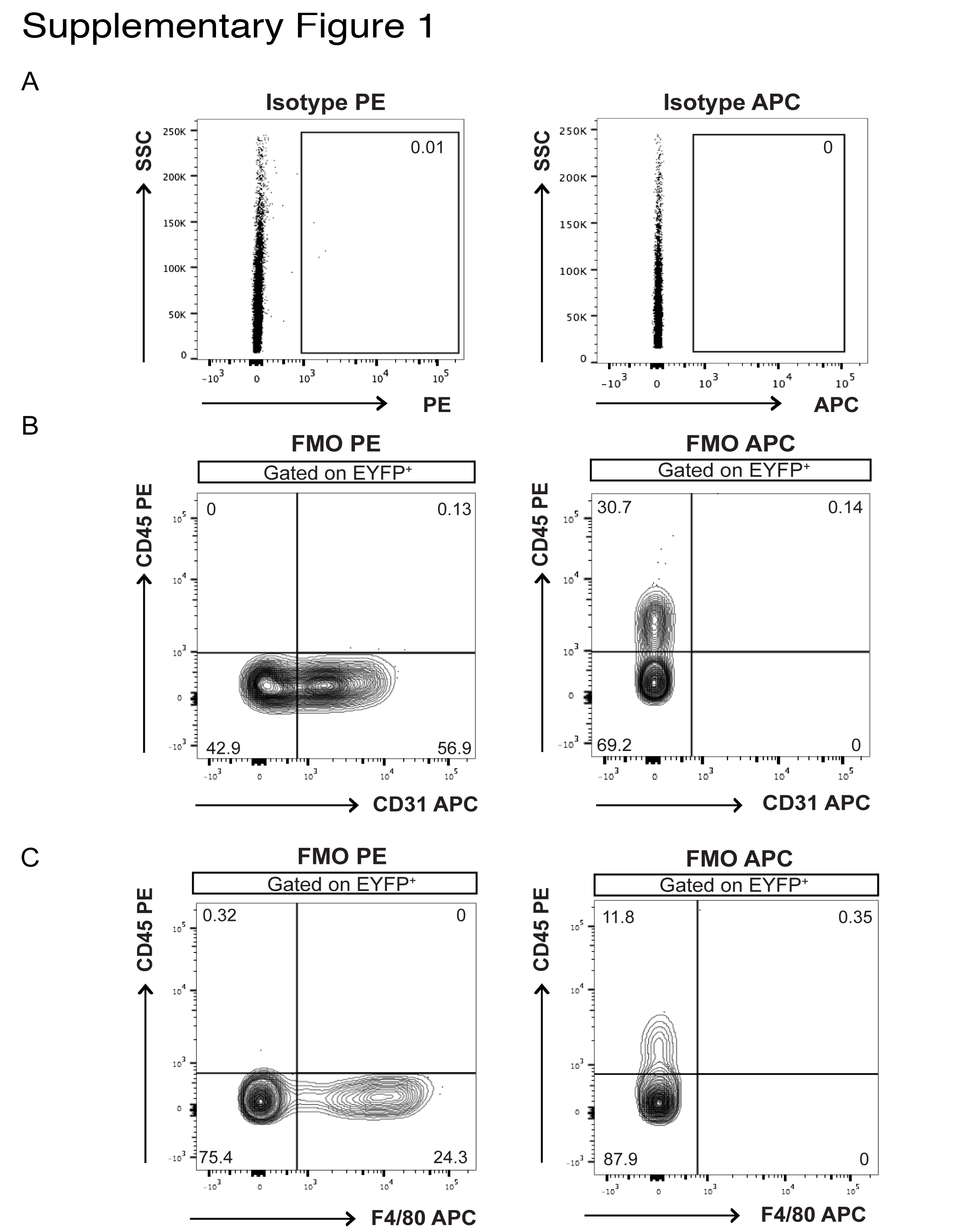

Supplement: S1 Fig — A) Anti-IgG2b,k- PE and Anti-IgG2a,k-APC isotype controls for FACS analysis of E12.5 embryos. B) Representative density plots showing FMO controls for FACS analysis of E12.5 cells. Plots show gating of CD31 and CD45 within the EYFP+ population. C) Representative density plots showing FMO controls for FACS analysis of E12.5 cells. Plots show gating of CD31 and F4/80 within the EYFP+ population. (TIF) [file pone.0164893.s001.tif]

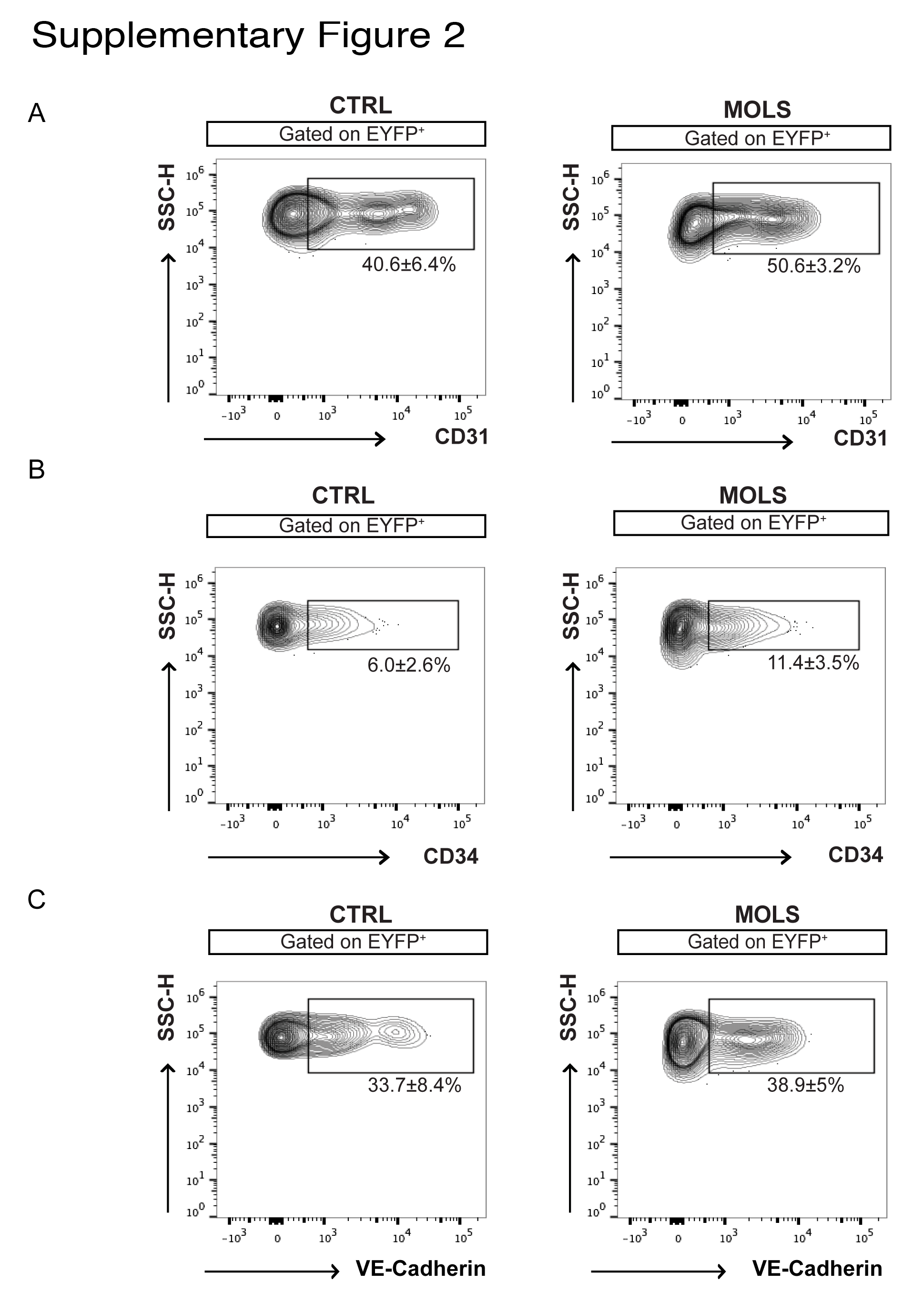

Supplement: S2 Fig — A-C) Representative FACS plots of E12.5 embryos showing the percentage of (A) CD31, (B) CD34 and (C) VE-Cadherin within the EYFP+ subset in control and molsidomine treated embryos. Data are expressed as mean ± S.E.M. (At least n = 3 embryos per group). (TIF) [file pone.0164893.s002.tif]
