# Supplementary material for: Nitric Oxide Donor Molsidomine Positively Modulates Myogenic Differentiation of Embryonic Endothelial Progenitors
Source: PLoS One. 2016 Oct 19;11(10):e0164893. doi: 10.1371/journal.pone.0164893 (PMC5070765; doi:10.1371/journal.pone.0164893)
Supplement: S1 Table — (PDF) [file pone.0164893.s003.pdf]

S1 Table. Antibodies used for Immunofluorescence.

Primary Antibodies				
**Antibody**	**Host**	**Dilution**	**Clone**	**Supplier**
Myosin Heavy Chain	Mouse	1:2	MF20	DHSB
MyoD	Mouse	1:50	5.8A	DAKO
GFP	Rabbit	1:300	Polyclonal	Life Technologies
GFP	Mouse	1:100	3E6	Life Technologies
CD31 / PECAM1	Rat	1:2	MEC13.3	Gift from E. Dejana

Secondary Antibodies			
**Antibody**	**Host**	**Dilution**	**Supplier**
Anti-Rabbit Alexa 488	Donkey	1:500	Molecular Probes
Anti-Rat Alexa 546	Goat	1:500	Molecular Probes
Anti-Rat Alexa 647	Chicken	1:500	Molecular Probes
Anti-Mouse Alexa 488	Goat	1:500	Molecular Probes
Anti-Mouse Alexa 546	Goat	1:500	Molecular Probes
