# Supplementary material for: Nitric Oxide Donor Molsidomine Positively Modulates Myogenic Differentiation of Embryonic Endothelial Progenitors
Source: PLoS One. 2016 Oct 19;11(10):e0164893. doi: 10.1371/journal.pone.0164893 (PMC5070765; doi:10.1371/journal.pone.0164893)
Supplement: S2 Table — (PDF) [file pone.0164893.s004.pdf]

**S2 Table. Antibodies used for FACS analysis and FACS sorting.**

| **Primary Antibodies and Conjugates** | | | | |
|---|---|---|---|---|
| **Antibody** | **Host** | **Dilution** | **Clone** | **Supplier** |
| F4/80-APC | Rat | 1:100 | CI:A3-1 | Abd Serotec |
| CD45-PE | Rat | 1:100 | 30-F11 | BD |
| CD31-APC | Rat | 1:100 | MEC13.3 | BD |
| CD31-PE | Mouse | 1:100 | 390 | eBioscience |
| CD34-Alexa 647 | Mouse | 1:100 | RAM34 | Biolegend |
| VE-Cadherin-APC | Rat | 1:100 | BV13 | eBioscience |
| IgG2a,k-APC | Rat | 1:100 | eBR2a | eBioscience |
| IgG2b,k-PE | Rat | 1:100 | MPC-11 | BD |
