# Supplementary material for: Nitric Oxide Donor Molsidomine Positively Modulates Myogenic Differentiation of Embryonic Endothelial Progenitors
Source: PLoS One. 2016 Oct 19;11(10):e0164893. doi: 10.1371/journal.pone.0164893 (PMC5070765; doi:10.1371/journal.pone.0164893)
Supplement: S3 Table — (PDF) [file pone.0164893.s005.pdf]

S3 Table. Primers used for quantitative real-time PCR.

**Gene**	**Forward primer**	**Reverse primer**
28S	AAACTCTGGTGGAGGTCCGT	CTTACCAAAAGTGGCCCACTA
cyclophillin A	CATACGGGTCCTGGCATCTTGTCC	TGGTGATCTTCTTGCTGGTCTTGC
CD31	AGGGGACCAGCTGCACATTAGG	AGGCCGCTTCTCTTGACCACTT
CD34	ACCACACCAGCCATCTCAG	TAGATGGCAGGCTGGACTTC
Desmin	AATAAGAACAACGATGCGCTG	CTGGCTTACAGCACTTCATGT
MyoD	AATAAGAACAACGATGCGCTG	CTGGCTTACAGCACTTCATGT
Myogenin	GACATCCCCCTATTTCTACCA	GTCCCCAGTCCCTTTTCTTC
eMHC	TGAAGAAGGAGCAGGACACCAG	CACTTGGAGTTTATCCACCAGATCC
Pax3	TGAGTTCTATCAGCCGCATC	GCCTTTTTCTCGCTTTCTTC
VE-Cadherin	GTACAGCATCATGCAGGGCG	ATTCGTATCGGATAGTGGGG
